# Infections

**Published:** 1916-10

**Authors:** 


### Infections.

**Typhoid Reactions in the Testicle, Epididymis and Spermatic Cord.** A. Satre alludes to a previous report of Olmer & Voisin on several cases of typhoid orchi-epididymitis, whose occurrence is about 1:200 cases of fever. He reports 5 cases of reaction in one or more of these organs following antityphoid inoculation. He considers the reaction independent of venereal or rheumatic infection in the past, even if such is shown in the history, and too rapid in its occurrence (as within 24 hours) to be an inflammation. He has noted 28 cases in 2900 inoculations, the combined inoculation against typhoid and A and B paratyphoid producing the reaction more frequently than either alone. Mild revulsive treatment, pyramidon and suspension reduced the swelling and pain quite promptly.

